# The management of neuropathic ulcers of the foot in diabetes by shock wave therapy

**DOI:** 10.1186/1471-2474-10-54

**Published:** 2009-05-27

**Authors:** Biagio Moretti, Angela Notarnicola, Giulio Maggio, Lorenzo Moretti, Michele Pascone, Silvio Tafuri, Vittorio Patella

**Affiliations:** 1 Department of Clinical Methodology and Surgical Techniques, Orthopedics Section, Faculty of Medicine and Surgery of University of Bari, General Hospital, Piazza Giulio Cesare 11, 70124 Bari, Italy; 2 Plastic Surgery Unit, Faculty of Medicine and Surgery of University of Bari, General Hospital, Piazza Giulio Cesare 11, 70124 Bari, Italy; 3 President of Course of Motor and Sports Sciences, Faculty of Medicine and Surgery of University of Bari, General Hospital, Piazza Giulio Cesare 11, 70124 Bari, Italy; 4 Hygiene Section, Department of Biomedical Sciences and Human Oncology, Faculty of Medicine and Surgery of University of Bari, General Hospital, Piazza Giulio Cesare 11, 70124 Bari, Italy; 5 *SITOD*, Italian Society of Shock Waves Therapy, Naples, Italy

## Abstract

**Background:**

Diabetes is becoming one of the most common chronic diseases, and ulcers are its most serious complication. Beginning with neuropathy, the subsequent foot wounds frequently lead to lower extremity amputation, even in the absence of critical limb ischemia. In recent years, some researchers have studied external shock wave therapy (ESWT) as a new approach to soft tissue wound healing. The rationale of this study was to evaluate if ESWT is effective in the management of neuropathic diabetic foot ulcers.

**Methods:**

We designed a randomized, prospective, controlled study in which we recruited 30 patients affected by neuropathic diabetic foot ulcers and then divided them into two groups based on different management strategies. One group was treated with standard care and shock wave therapy. The other group was treated with only standard care. The healing of the ulcers was evaluated over 20 weeks by the rate of re-epithelization.

**Results:**

After 20 weeks of treatment, 53.33% of the ESWT-treated patients had complete wound closure compared with 33.33% of the control patients, and the healing times were 60.8 and 82.2 days, respectively (p < 0.001). Significant differences in the index of the re-epithelization were observed between the two groups, with values of 2.97 mm^2^/die in the ESWT-group and 1.30 mm^2^/die in the control group (p < 0.001).

**Conclusion:**

Therefore, ESWT may be a useful adjunct in the management of diabetic foot ulceration.

**Trial registration:**

Current Controlled Trials ISRCTN21800909

## Background

The rapid rise in the incidence of diabetes is an alarming concern to health care professionals, largely because of the serious associated complications. Recent data from the Centers for Disease Control and Prevention approximate that 20.8 million people, roughly 7% of the United States' population, have diabetes [1]. In 2005 alone, 1.5 million new cases of diabetes were diagnosed in people aged 20 years or older [1]. Diabetes mellitus is a disease known for its multifaceted complications, and foot ulceration, which often results in lower extremity amputations, is one of the most common complications associated with the disease [2-5]. The prevalence of foot ulcers ranges from 4% to 10% among persons diagnosed with diabetes [6]. This translates to an annual population-based incidence of 1.0% to 4.1% and a lifetime incidence as high as 25% [6].

At least 15% of people with diabetes will eventually develop a lower-extremity ulcer of some sort [7]. Foot deformities and limited joint mobility impose excessive pressure on the plantar portion of the foot. This limitation in joint mobility is secondary to non-enzymatic glycosylation of the periarticular soft tissues, and it reduces the foot's ability to accommodate for ambulatory ground reactive force to increase plantar pressure [8-13]. This excessive pressure, combined with the repetitive or constant stress from daily ambulation along with neuropathy, will ultimately lead to failure of the protective integument and ulceration. Although the precise pathophysiological mechanism underlying the development of diabetic foot ulcerations is complex [13], it is generally associated with the presence of peripheral neuropathy and repetitive trauma due to normal walking activities which expose the foot to moderate or high pressure and shear forces [14-16]. Brand [17] theorized that a local inflammatory response, focal tissue ischemia, tissue destruction and ulceration may occur when these types of forces are applied to a specific area over an extended period of time. Ulceration sites correlate with the highest plantar pressure points [18-22]. By definition, these ulcers present within the context of arterial perfusion adequate for wound healing [23]. The current standard treatment for foot ulcers consists of debridement, treatment of infection, pressure relief and arterial revascularization, if required [24].

The main objective of the present study was to evaluate the healing rates of diabetic foot ulcers during a 20-week period in patients treated with ESWT (plus standard therapy), compared with standard therapy consisting of debridement and Silvercell dressing.

## Methods

This study is a randomized, prospected, controlled, clinical trial. A total of 30 patients were recruited from the Diabetic Ambulatory of Endocrinology Unit of the University of Bari (Italy) between October 1, 2006 and March 31, 2007. Study subjects were evaluated for a total of 20 weeks.

Inclusion criteria were neuropathic foot plantar ulceration below the malleoli for a period of at least 6 months with an area wider than 1 cm^2^, age 30–70 years, a diameter of the lesion between 0.5 and 5 cm and type 1 diabetes mellitus with insulin treatment for at least 5 years prior. Patients also should have had peripheral neuropathy, as defined by insensitivity to a 10-g monofilament and by a vibration perception threshold measured at the malleolus of at least 25 volts [25]. The vascular assessment consisted of an ankle-brachial index > 0.7 and palpation of the dorsalis pedis and posterior tibial arteries. If one or both arterial pulses were not palpable, the subject was excluded.

Exclusion criteria included patients with any of the following around the time of ESWT applications: peripheral vascular disease, coronary bypass, pregnancy, coagulation diseases or history of neoplasia or other conditions, based on the principal investigator's clinical judgment.

The population was randomized into two groups that received standard care consisting of therapeutic footwear, debridement and dressing. In addition to the standard therapy, the ESWT group also received three applications of shock wave therapy.

The study protocol and informed consent were approved by the Ethics Committee of the University of Medicine in Bari (Italy). Usual clinical management for neuropathic ulcers was used, that is a regular debridement to remove surrounding callus and local would care by Silvercell dressing for an average of 48–72 hours. Then, in the ESWT group, the shock wave applicator head was placed over the wound, utilizing ultrasonic gel and plastic draping to prevent any cross-contamination of the device.

The treatment lasted just one or two minutes. The protocol consisted of a course of three sessions (every 72 hours), with 100 pulses per 1 cm^2 ^of wound delivered at each session at a flux density of 0.03 mJ/mm^2 ^using a electromagnetic lithotripter (MINILITH SL1 by STORZ MEDICAL) with a cylindrical coil, parabolic focus and ultrasound scanning. We aimed the device directly around the perimeter of the ulcer. The most the patient may have noticed was the sound of the ESWT machine as it generates the shock wave, so no local anesthetic was used during treatment.

Patients in the control group were treated with the essentials of foot ulcer care, namely debridement, adequate pressure relief and treatment of infection, as required by current international guidelines [24].

Patients were permitted to ambulate as tolerated, and each patient was provided with an orthopedic device to remove mechanical stress and pressure at the site of the ulcer during walking.

The ulcers were photographed by digital camera using the macro function. The wound area and its following reductions were measured with the Rhinoceros program running on a personal computer.

If clinical signs of infection were evident, a swab for bacteriological analysis was taken, and the microbiological results were recorded. Wound infection was treated by appropriate systemic antibiotics.

All data elements recorded during the study period were entered and validated in Microsoft Excel. The time to complete ulcer healing was measured as the number of days from the start of treatment to the date in which each patient achieved complete wound healing. If the healing did not occur within the 20 weeks of the study, the patient was considered to be non-healing and the time was not registered. The comparison between the two treatment groups was made as the proportion of patients (%) who reached target healing of their ulcers at the end of the study. The time to complete healing and the index of re-epithelization of the wound area were compared between the two groups. All data were expressed as mean ± SD and analyzed by Student's t-test. P value less than 0.05 was regarded as significant.

## Results

There were no significant differences between the two groups in terms of demographics and clinical data, as reported in tables 1 and 2. Group A (treated with ESWT) was composed of fifteen patients: nine males and six females. The age of these patients was 56.2 +/- 4.9 (mean +/- DS). Group B (treated with standard management) was composed of fifteen patients: seven males and eight females. The age of all the patients was 56.8 +/- 7.5 (mean +/- DS). The median size of the lesion in group A was 297.8 +/- 129.4 mm^2 ^(mean +/- DS) and in group B was 245 +/- 100.9 mm^2 ^(mean +/- DS).

**Table 1:** Characteristics of patients in the ESWT group (group A)

**Patient**	**Age**	**Initial area (mm^2^)**	**Complete healing (mm^2^)**	**Re-epithelization index (mm^2^/die)**
1. woman	47	185	60	3.1
2. man	50	390	-	2.7
3. man	66	440	-	3
4. woman	55	180	62	2.9
5. man	61	205	66	3.1
6. man	50	400	-	2.7
7. man	58	145	60	2.4
8. woman	62	450	-	2.9
9. woman	53	175	52	3.4
10. woman	56	180	66	2.7
11. man	55	460	-	3.2
12. man	56	215	57	3.8
13. man	58	375	-	2.6
14. man	57	187	64	2.9
15. woman	60	480	-	3.2

**Table 2:** Characteristics of patients in the control group (group B).

**Patient**	**Age**	**Initial area (mm^2^)**	**Complete healing (mm^2^)**	**Re-epithelization index (mm^2^/die)**
1. man	45	440	-	1.3
2. man	55	145	80	1.8
3. man	43	300	-	1.5
4. woman	58	145	90	1.6
5. woman	63	250	-	1.3
6. man	59	275	-	1.2
7. woman	57	290	-	1.1
8. woman	68	350	-	0.7
9. man	52	100	83	1.2
10. woman	63	300	-	1.4
11. woman	65	105	80	1.3
12. man	59	310	-	1
13. woman	52	250	-	1.4
14. man	48	116	78	1.5
15. woman	65	300	-	1.2

All patients of both groups completed the study and attended all control visits. No significant differences emerged between the two groups with regard to treatment complications.

The proportions of ulcers that healed in 20 weeks in the A and B groups were 53.33% and 33.33%, respectively.

For the ulcers that healed during the 20-week period, the healing times were 60.8 +/- 4.7 days (mean +/- DS) in group A and 82.2 +/- 4.7 days (mean +/- DS) in group B patients (p < 0.001).

**Figure 1:**
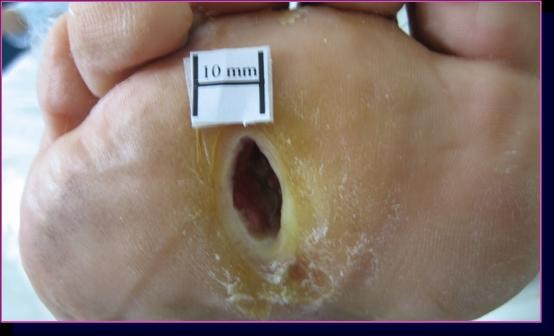
**The ulcer of case 9 in group A**. The lesion was localized to the plantar surface of the 3^rd ^intermetatarsal space and was 175 mm^2 ^before ESWT.

**Figure 2:**
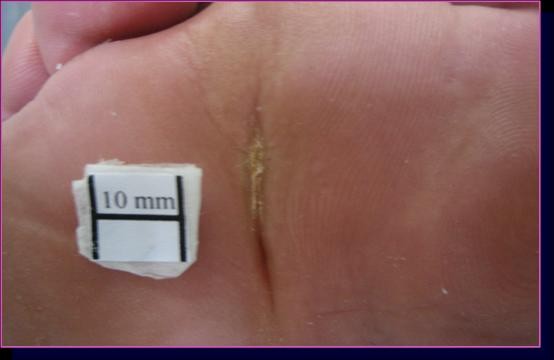
**Ulcer of case 9 in group A**. The lesion healed after 52 days with a re-epithelization index of 3.4 mm^2^/die.

**Figure 3:**
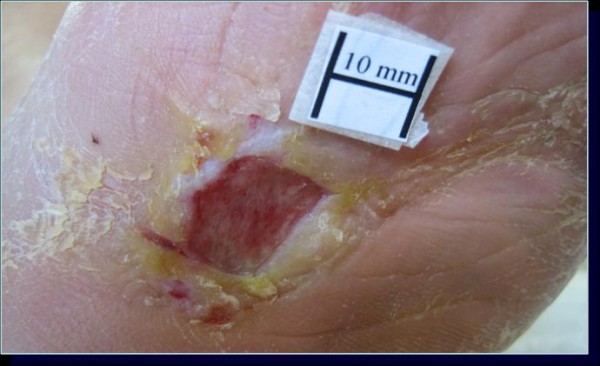
**Ulcer of case 4 in group B**. The lesion was localized to the plantar surface of the tarsus and was 145 mm^2 ^before standard management.

**Figure 4:**
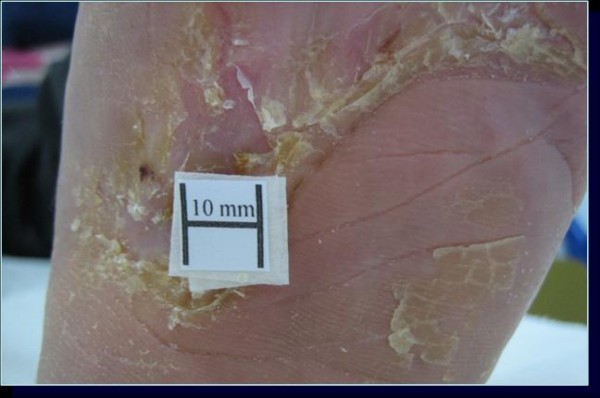
**Ulcer of case 4 in group B**. The lesion healed after 90 days with a re-epithelization index of 1.6 mm^2^/die.

A significant difference was observed in the index of the re-epithelization between the two groups, with values of 2.97 +/- 0.34 mm^2^/die (mean +/- DS) in the ESWT group [fig. 1 and 2] and 1.30 +/- 0.26 mm^2^/die (mean +/- DS) in the control-group (fig. 3 and 4) (p < 0.001). Both the healing rate and the healing time were increased in the ESWT group, and the differences were statistically significant.

One patient in each group developed local signs of infection (peri-lesional erythema and edema), which led to the administration of oral antibiotics (1 g b.i.d. amoxicillin-clavulanate) for 10 days. The signs of infection were resolved in five days in the one patient in the ESWT group and in one week in the patient in the control group, and both patients remained in the study.

## Discussion

Neuropathic foot ulcers generally do not respond well to treatment, and several novel treatment modalities have been proposed over the past few years [26-29], including the development of new dressings, growth factors, bioengineered skin and tissue substitutes, hyperbaric oxygen, negative pressure wound therapy and other novel approaches to stimulate wound healing [30,29-33]. Limbs with non-healing neuropathic ulcers may eventually require amputation. Individuals with lower-limb amputations are at risk for developing concomitant medical ailments, report a diminished quality of life and are more likely to die than other individuals with diabetes [34]. In a meta-analysis of standard ulcer treatment, only 30% of individuals with a neuropathic diabetic foot ulcer will heal within 20 weeks of commencing good care [35].

For the past 20 years, ESWT has been used in the treatment of a number of musculoskeletal conditions, including plantar fasciitis, tendinosis calcarea of the shoulder, tennis elbow, pseudoarthrosis and algodystrophy. A shock wave (SW) is a longitudinal acoustic wave, travelling with ultrasonic speed in the water of the body tissue, which is a single pressure pulse with a short needle-like positive spike of less than 1 microsecond with a lower amplitude [36]. SWs are known to exert "cavitation effects" (a micrometer sized violent collapse of bubbles inside the cells) and have recently been demonstrated to induce localized impulses on cell membranes that resemble shear stress [36,37]. The rational of this treatment is the stimulation of tissue healing, reduction of calcification and inhibition of pain receptors or denervation to achieve pain relief [38-40].

Researchers have shown that the local delivery of shock wave therapy stimulates the early expression of angiogenesis-related growth factors, including endothelial nitric oxide synthase, vascular endothelial growth factor and proliferating cell nuclear antigen. As such, it results in new vessel in-growth that improves blood supply, increases cell proliferation and accelerates tissue regeneration and healing [41,42]. A study of ESWT application on the porcine heart indicates that low-energy ameliorates myocardial perfusion and cardiac function in a model of chronic myocardial ischemia, and clinical trials started in patients with severe chronic artery disease and no adverse effects were found [43,44]. Recently, this treatment has also been applied to skin lesions. In a previous study [45], treatment with ESW was demonstrated to enhance epigastric skin flap survival in rats, as confirmed by the significant reduction in necrotic flap zones. Additionally, in tissue samples adjacent to the necrosis areas, increased vascular endothelial growth factor expression was observed in the ESW-treated skin flap [46]. Histological staining indicated that ESW treatment substantially increased vascular endothelial growth factor and proliferating cell nuclear antigen expression, reduced leukocyte infiltration and suppressed tumor necrosis factor alpha expression in flap tissue ischemic zones compared with controls. It was postulated that ESW treatment has a positive effect in rescuing the ischemic zone of flaps by increasing tissue perfusion, and it is associated with a suppression of the inflammatory response [47]. After experimental studies in animal models, the clinical application of shock waves for the therapy of acute and chronic soft tissue wounds is becoming more popular. Two hundred and eight patients were prospectively enrolled into a trial, and the treatment consisted of debridement and ESWT at 100 to 1,000 shocks/cm^2 ^at 0.1 mJ/mm^2^. Since surface defects are often involved, researchers modified the shock wave head so that the shock wave would no longer be focused in a small plane of the treatment area. It was found that 75% of treated patients had 100% wound epithelization during a 3 to 12 week period of monitoring [48].

**Figure 5:**
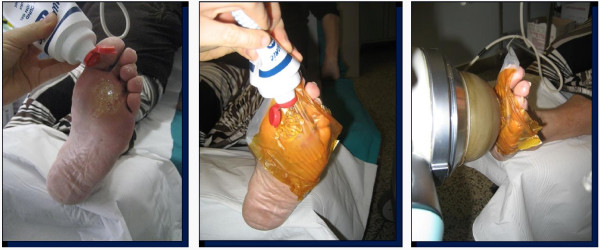
**The steps of ultrasonic gel, plastic draping and shock wave administration at work in the study**.

Here we have completed a clinical trial to evaluate the possibility of utilizing this treatment for wounds that have a difficult recovery. We used an electromagnetic generator, which we apply in the treatment of the orthopedic diseases (fig. 5). We selected patients affected by diabetic neuropathic foot ulcers. In a detailed analysis by Margolis et al [35], of the factors that may contribute to healing, the only ones that emerged from logistic regression were ulcer area, ulcer duration and the race of the patient. This study revealed that those patients with a diabetic neuropathic foot ulcer that healed within 20 weeks using standard care were more likely to have a smaller wound that existed for a shorter period and to be non-whites, compared with patients whose wounds did not heal within 20 weeks. The neuropathic ulcers we studies were present for a longer time prior to treatment, became larger and took more time to heal. The patient's age, serum level of glycosylated hemoglobin at the start and sex were not associated with the probability of wound healing. Nevertheless, no difference was observed in the rate of healing of plantar and non-plantar ulcers. In the meta-analysis, only 30.9% of the diabetic neuropathic ulcers healed after 20 weeks of good treatment [35].

During our previous preliminary experience in the application of the shock waves for the treatment of ulcers, we found that the rate of re-epithelization was higher in big ulcers. To avoid any selection bias, we chose a homogeneous type of ulcer that was smaller than 5 cm in diameter and of non-recent arising, both plantar and dorsal and in Caucasian patients. In according with Ethic Committee's indications, we ensured an advanced dressing in the management of the ulcers. Silvercell satisfied the need of antimicrobial action and of exudates management in all the ulcers of the study.

Our trial is the first randomized, controlled clinical study on shock wave therapy for diabetic foot ulcers, and it shows promising results for the application of ESWT [fig. 5]. Statistically significant differences were demonstrated in the index of re-epithelization. It provides evidence that ESWT doubles the mean healing index of re-epithelization in diabetic, non-ischemic, chronic foot ulcers. In addition, it suggests the possibility of shortening the time of healing.

In our two cases of infection we observed a rapid resolution in the SW group. The observed antibacterial effects of the extracorporeal shockwaves could be highly relevant for the treatment of non-healing wounds, which are commonly at increased risk of infection [49].

The rationale for the use of ESWT as an adjunctive treatment for the diabetic foot arises from its beneficial effects on the microenvironment of the wound. It stimulates physiological angiogenesis, due to the release of NO and vascular growth factors at the site of the ulcer. Recent results suggest that SW therapy could be effective and safe for the treatment of peripheral artery disease [50]. In a rabbit hindlimb ischemia model, the development of collateral arteries, the flow ratio of the ischemic/non-ischemic common iliac arteries, the blood pressure ratio of the ischemic/non-ischemic hindlimb and the capillary density in the ischemic muscles were all significantly increased in the SW group at three weeks after therapy compared with the control group. These results indicate that the SW therapy induced therapeutic angiogenesis. Importantly, no adverse effects, such as muscle damage, hemorrhage or thrombosis, were noted with the therapy, and the expression levels of eNOS and VEGF proteins tended to be increased.

We administrated the shockwaves every 72 hours for 3 sessions. We chose this protocol on the basis of our clinical experience in Orthopedics treatment. Recently, the International Society for Medical Shockwaves Treatment published the *"New guidelines for ESWT" *and it suggested to apply from 1 to 6 sessions, using an interval of 1 week [51]. However, in literature good results of angiogenesis are reported using different protocols in which the interval between each session of shock waves varies from 48 hours to two weeks [43,52]. Following studies should be useful for compare the effects of protocols which should be different only for the interval time between each treatment or for the dosage of shock waves.

ESWT seems effective in accelerating the healing rate of non-ischemic chronic diabetic foot ulcers. Our new results reinforce the interest in applying ESWT to ulcers associated with neuropathy and macroangiopathy. On the basis of these results, we can hypothesize that ESWT should also be valid for arteriogenic ulcers, and we are planning a new clinical trial to evaluate the effects of ESWT on this type of ulcer.

## Conclusion

The present study assessed the safety and efficacy of shock waves for the treatment of diabetic ulcers. There is a high occurrence of foot ulcers within the population of diabetics. Foot ulcerations may lead to lower extremity amputations and are major causes of disability to patients, often resulting in significant morbidity, extensive periods of hospitalization and mortality. In order to diminish the detrimental consequences associated with diabetic foot ulcers, a high standard of care must be provided. In the present study, we have shown that the complete wound healing rate was significantly increased in ESWT-treated patients when compared with patients treated with the standard state of the art care available at present. This was accompanied by a significant reduction in the median time required to heal the ulcer with no increase in the rate of adverse reactions.

## Competing interests

The authors declare that they have no competing interests.

## Authors' contributions

BM made substantial contributions to the concept and design of the study. AN was involved in drafting the manuscript and critically revising it for intellectual content. GM made contributions to acquisition, analysis and interpretation of the data. LM carried out the studies, participated in ESWT treatment and ulcer management and performed the statistical analysis. MP conceived the study and participated in its design and coordination. VP participated in the design and coordination of the study and helped to draft the manuscript. All the authors read and approved the final manuscript.

## Pre-publication history

The pre-publication history for this paper can be accessed here:


